# The Epidemiology of Metacarpal Fractures: A Descriptive Study Based on 18,802 Fractures From the Swedish Fracture Register

**DOI:** 10.1016/j.jhsg.2025.02.015

**Published:** 2025-05-22

**Authors:** Jakob Deuschl, Henrik Alfort, Maria Wilcke

**Affiliations:** ∗ Department of Clinical Science and Education, Karolinska Institutet, Södersjukhuset, Stockholm, Sweden; † Department of Hand Surgery, Södersjukhuset, Stockholm, Sweden

**Keywords:** Epidemiology, Hand fractures, Metacarpal fractures, Metacarpals

## Abstract

**Purpose:**

Hand fractures are common injuries, but there are few large and detailed epidemiological reports describing them.

**Methods:**

This study describes the distribution of metacarpal fractures and their impact on patient-reported outcome measures based on 18,802 fractures from the Swedish Fracture Register.

**Results:**

The mean age at the time of injury was 39 years (men 33 years and women 52 years). The age distribution for women was bimodal. For men, there was unimodal peaking in early adulthood. Male injuries represented 70% of all metacarpal fractures. In men and children, the most common fracture was a distal extra-articular fracture of the fifth metacarpal, whereas in women, it was a shaft fracture of the fifth metacarpal. Fracture localization differed between metacarpals; 79% of the fractures in the first metacarpal were proximal, compared with 19% to 25% in the other metacarpals. Shaft fractures were the most common in the third and fourth metacarpals, and distal fractures were the most common in the second and fifth metacarpals. The most common cause of injury was a fall. Fractures of the first metacarpal were treated surgically to a greater extent than fractures in the other metacarpals. Men were operated on more often than women (19% vs 14%). Metacarpal fractures did not affect patient-rated hand function or quality of life 1 year after injury.

**Conclusions:**

Metacarpal fractures are common and mostly treated nonsurgically and have a minimal effect on patient well-being.

**Type of study/level of evidence:**

Therapeutic II.

Hand fractures are among the most common fractures in the upper extremity.^1^^,^^2^ Metacarpal fractures constitute a substantial part of hand fractures and affect patients of all ages.^2^, ^3^, ^4^, ^5^, ^6^, ^7^, ^8^, ^9^ Previous studies on the epidemiology of metacarpal fractures are either based on limited samples or do not distinguish between the different metacarpals.^3^^,^^9^, ^10^, ^11^, ^12^ There is, as of yet, no study that describes the fracture distribution among the five metacarpals and if and how treatment, age, and sex distribution differ between them. The impact of metacarpal fractures on patient-reported outcome measures (PROM) and quality of life has not been described.

This observational register study is based on data from the Swedish Fracture Register (SFR), a national quality register that collected data about fractures since 2011. The aim of this study was to describe the anatomical distribution of metacarpal fractures, their causes of injury, age and sex distribution, and treatment. We also investigated the impact of metacarpal fractures on PROM and self-assessed quality of life.

## Materials and Methods

All 54 trauma and orthopedic departments across Sweden are affiliated with the SFR. All fractures are registered by the treating physician according to the International Statistical Classification of Diseases (ICD-10), and Arbeitsgemeinschaft für Osteosynthesefragen/Orthopaedic Trauma Association (AO/OTA) classification based on the available radiographs. Demographic data of the patients are recorded, and patients fill out the Short Musculoskeletal Function Assessment (SMFA) and EuroQol 5 dimensions (EQ-5D) at baseline and 1 year after the injury.^13^, ^14^, ^15^, ^16^

**Figure 1:**
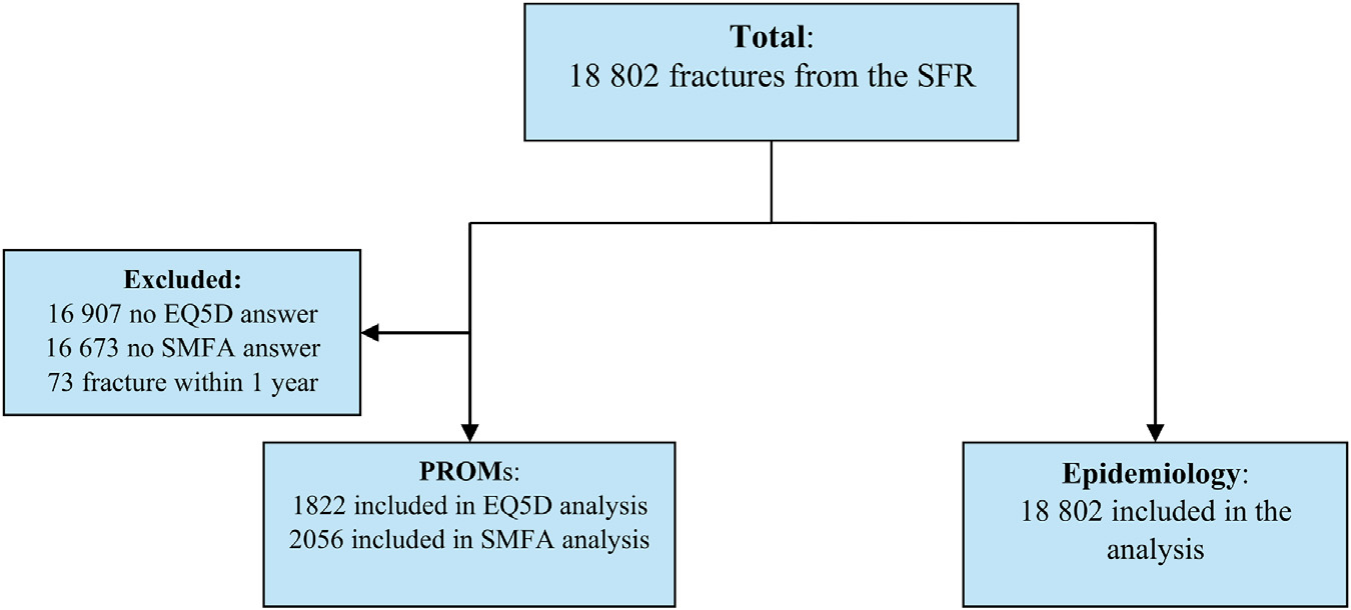
Flowchart of the patients.

Pseudonymized data was received on the 24th of June 2021 from the SFR for May 1, 2015 to December 31, 2019. Metacarpal fractures were identified with ICD-10 codes S.62.20-21 and S62.30-31. The data collected included fracture classification according to AO/OTA, age, sex, injury cause, treatment (surgical/nonosurgical treatment), and index scores for EQ-5D and SMFA arm/hand index before the injury and 1 year after the injury. High- or low-energy injury is determined by the registering physician according to SFR’s instructions stating that trauma such as traffic accidents, fall from height, workplace injuries involving machinery, and some sport activities are regarded as high-energy. A flowchart of the fractures is presented in Figure 1. For hand fractures, the national coverage rate, that is, the proportion of fractures registered in the SFR compared with the number of specific diagnoses registered in The National Patient Register, maintained by the National Board of Social Affairs and Health (Socialstyrelsen), was 35% in 2017, 35% in 2018, and 39% in 2019.^17^ Hand fractures in children are registered in SFR in the same way as in adults according to AO/OTA without consideration to affected physis.

### Fracture epidemiology

The fractures of the five metacarpals were categorized according to their AO/OTA classification as either a distal, shaft, or proximal fracture and as intra- or extra-articular. Patient age at the time of injury, cause and type of injury, anatomical distribution, and treatment for different metacarpal fractures were analyzed according to sex. Children were defined as patients under 16 years of age in the analysis.

### PROMs

The SMFA quantifies musculoskeletal dysfunction and contains 46 questions (Likert scales). The index is 0–100, and a higher score indicates more dysfunction and distress. The SMFA score is organized into six categories, of which the arm/hand function index (0–100) was used in this study. EuroQol 5 dimensions is a generic measure of the quality of life where 1 represents full health and 0 represents death. Children and patients who had not responded to both questionnaires were excluded from the analysis of PROMs (16,673 and 16,907 for SMFA and EQ-5D, respectively). Additionally, patients who sustained a new metacarpal fracture within the follow-up period (n = 73) were also excluded. This is because it would be unclear how each injury specifically impacted the PROMs.

### Statistics

Age is presented as mean and median (range). Patient-reported outcome measures scores are presented as median (interquartile range [IQR]). Changes in PROMs are presented as means (SD). The correlation between sex and treatment was analyzed with the χ^2^ test. Comparison of the individual change of PROMs from preinjury to 1 year post-injury was analyzed using the Wilcoxon signed-rank test (paired data). Differences in change in EQ-5D and SMFA arm/hand index from preinjury to 1 year post-injury between sexes, treatments, and fracture types were analyzed using the Mann-Whitney U test or the Kruskal-Wallis test (independent data). The significance level was set at *P* = .05. Correction for multiple testing was not performed.

The minimum clinically important difference (MCID) for EQ-5D is estimated to be 0.1.^18^ There is no reported MCID for the SMFA arm/hand index; hence, this was estimated with the distribution method, defining MCID as 0.5 SD of the calculated difference between the initial and follow-up index score for all fractures.^19^

### Ethical approval

Ethical approval had been obtained from the Swedish Ethical Review Authority (Reference number 2020-02115). Patient inclusion in a national quality register such as the SFR is regulated by Swedish legislation and approved by the Swedish Data Inspection Board. All patients with a fracture receive information about the register and are included unless they oppose it, and no signed consent is needed to use the data for research purposes.

## Results

### Fracture epidemiology

**Figure 2:**
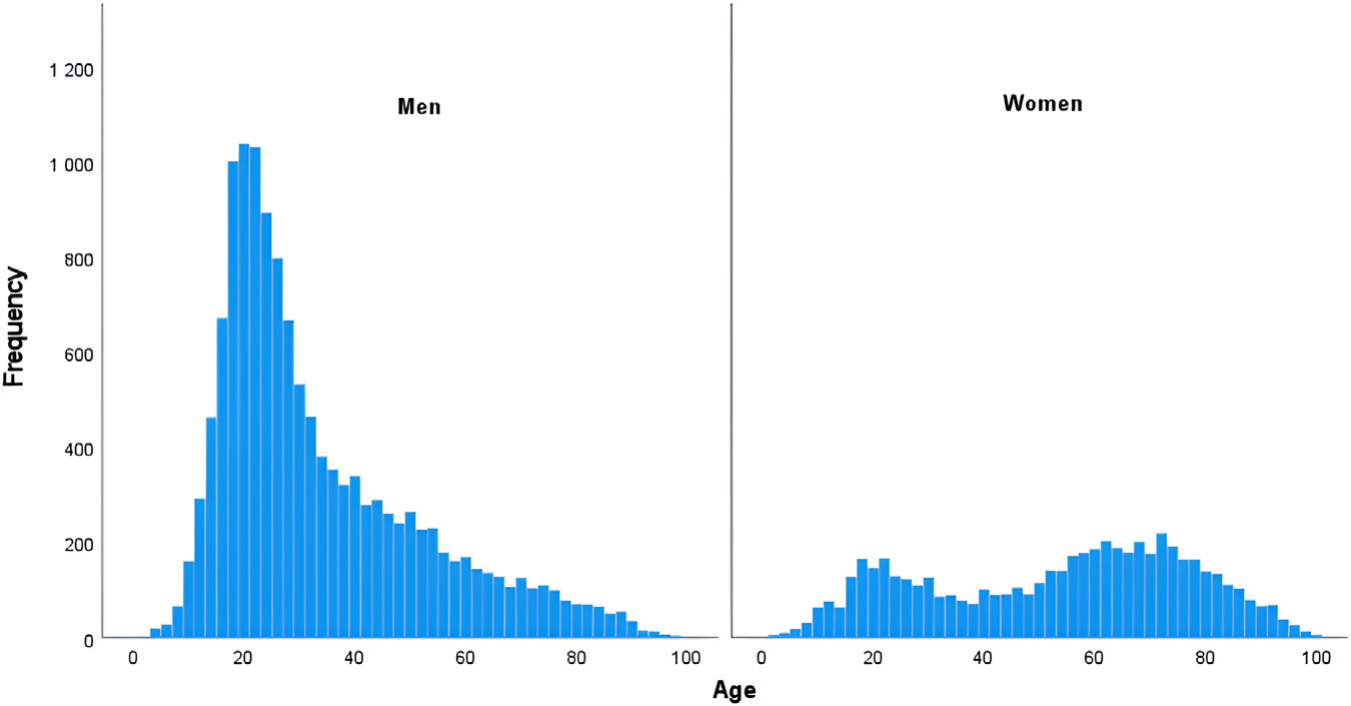
Age distribution of metacarpal fractures in men and women.

In total, 18,802 fractures in 16,573 patients were included (Fig. 1). The mean age was 39 years, and the median age was 32 years at the time of injury (range: 0–108). The mean age for men was 33 years (median age: 27 years, range: 0–98) and for women, was 52 years (median: 57 years, range: 1–108). Men experienced 70% of all metacarpal fractures. The age distribution was bimodal in women and unimodal in men (Fig. 2).

**Figure 3:**
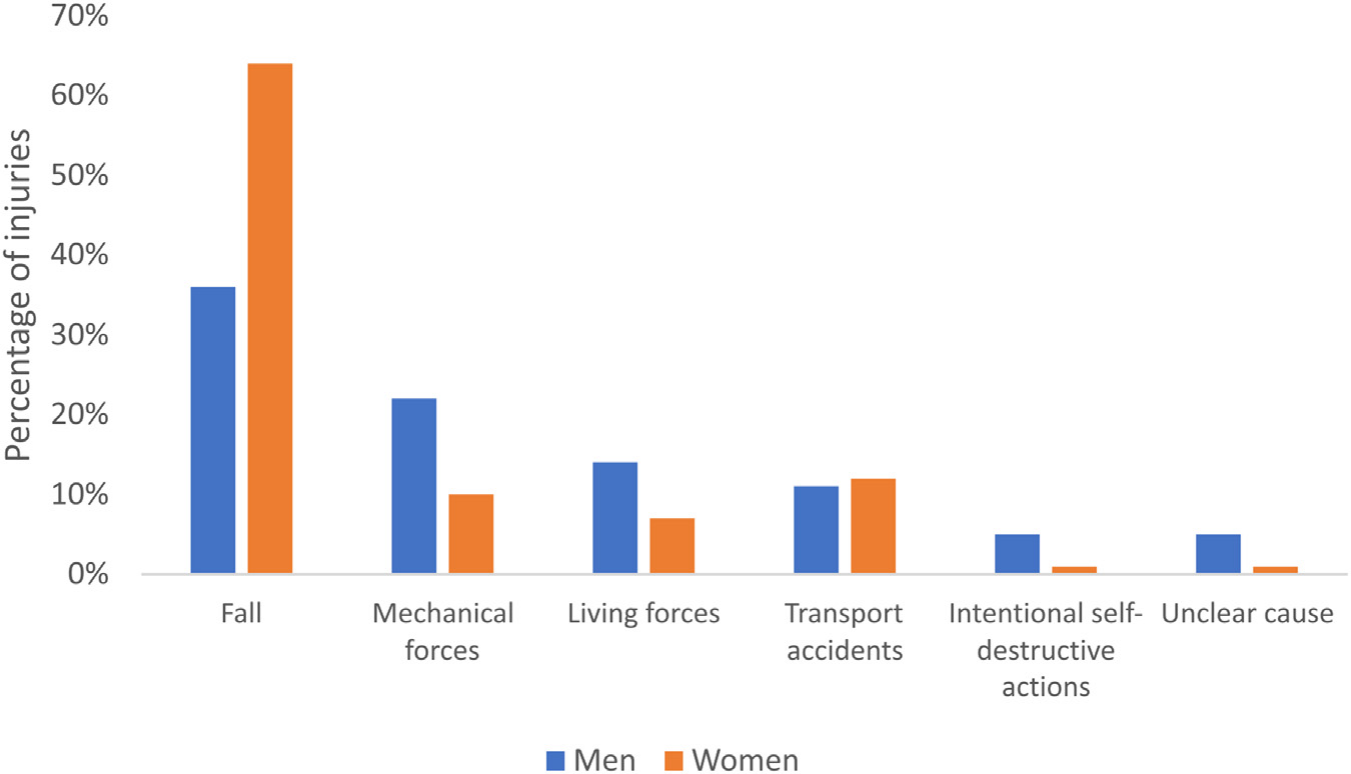
Injury causes. Presented as percentage of total injuries within each injury cause. Punching another person is a living force. Punching into an object is a mechanical force.

There was a reported cause of injury in 18,106 (96%) fractures. The most common cause of injury was a fall (Fig. 3). High-energy injuries were more common in men (10% vs 6%, *P* = .016). Fractures caused by high-energy injuries were treated surgically to a greater extent than low-energy injuries (30% vs 16%, *P* < .001) in both men and women. The same applied to open fractures, and 54% were treated surgically.

Table 1 presents the mean age, sex distribution, intra-/extra-articular fracture, and treatment in the different metacarpals. The fifth metacarpal accounted for 57% of all metacarpal fractures, followed by the fourth, first, third, and second. Fractures of the first metacarpal were treated surgically to a greater extent than in the other metacarpals. Open fractures represented 1.5% of all injuries and were more common in men (1.7% vs 1.2%, *P* = .031)

**Table 1:** Distribution of Metacarpal Fractures and Their Mean Age, Sex distribution, Open or Closed Fracture, and Treatment

Metacarpal	N	Percentage of Total Fractures (%)	Mean Age (y)	Percentage Within Men (%)	Percentage Within Women (%)	Open Fracture (%)	Intra-Articular Fracture (%)	Operative Treatment (%∗)
First	2,025	11	38	11	9	2	42	36
Second	1,238	7	37	7	6	5	23	18
Third	1,486	8	39	8	9	3	14	13
Fourth	3,285	17	41	17	20	1	12	18
Fifth	10, 768	57	39	57	56	1	16	14
All fractures	18,802	100	39	100	100	1.5	18	17

∗ 17,825 patients had a treatment method recorded.

**Figure 4:**
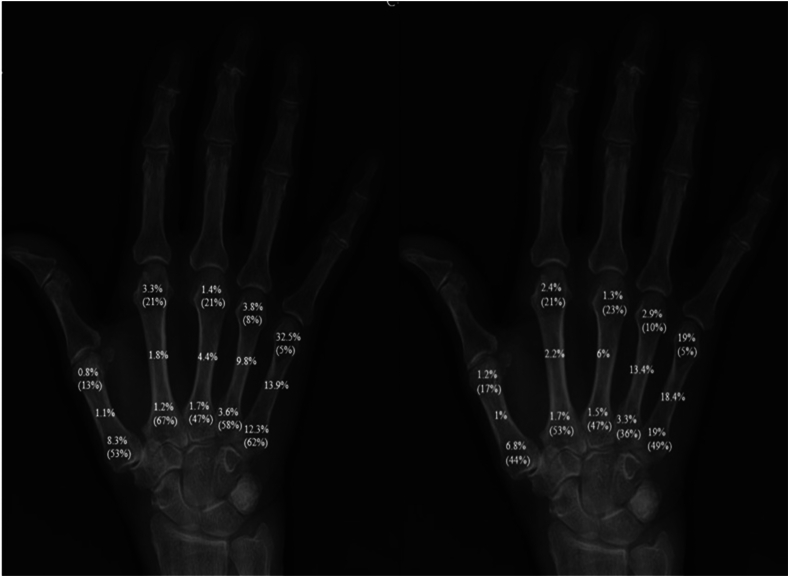
Fracture localization for metacarpal fractures in men (left) and women (right) of all ages. Percentages within parenthesis is the proportion of intra-articular fractures for each localization. Exact numbers of patients are presented in supplementary Table 1.

Men were treated surgically to a greater extent than women (19% vs 14%, *P* = <.001). Fracture location differed between the metacarpals; 74% of the fractures in the first metacarpal were proximal, compared with 19% to 25% in the other metacarpals. In the third and fourth metacarpals, shaft fractures were the most common, whereas distal fractures were the most common in the second and fifth metacarpals. In men, the most common fracture was a distal extra-articular fracture of the fifth metacarpal, whereas in women, it was a shaft fracture of the fifth metacarpal. The anatomical fracture distribution is shown in Figure 4 and Appendix S1 (available online on the Journal’s website at https://www.jhsgo.org). Intra-articular fractures were more frequently observed proximally rather than distally in the metacarpals.

**Figure 5:**
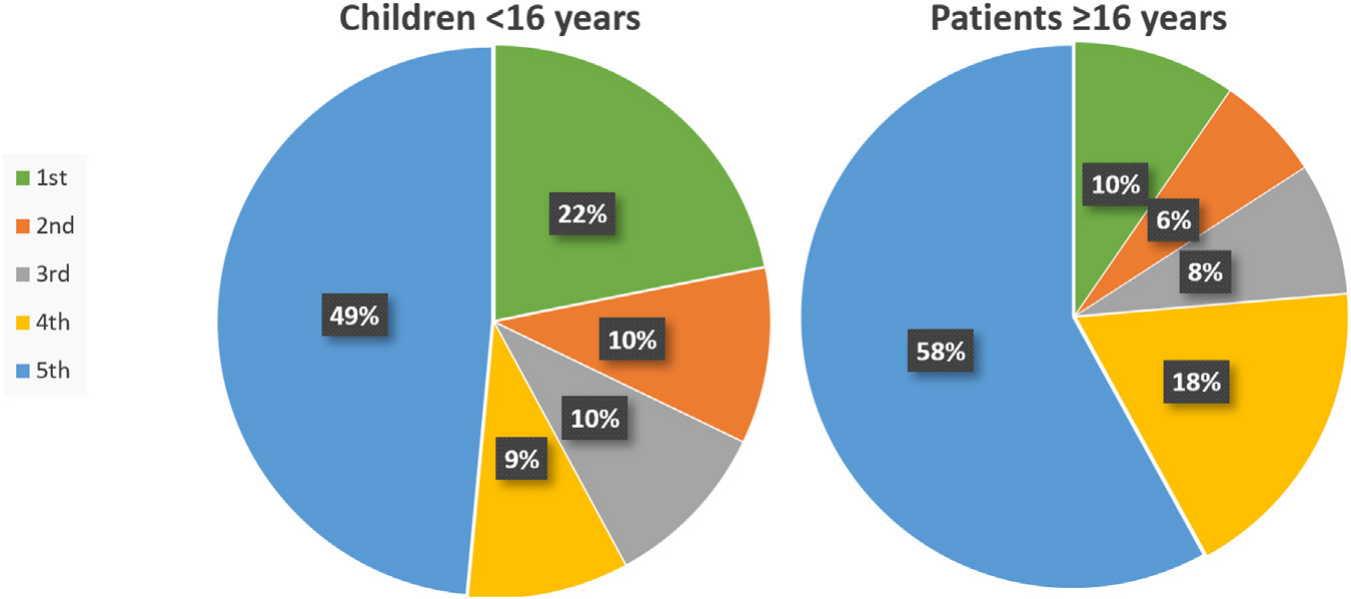
Distribution of metacarpal fractures. Children <16 years of age (left, n = 1,666) and patients ≥16 years of age (right, n = 17,136).

The distribution of fractures between the different metacarpals differed between children and adults (Fig. 5).

### PROMs

In total, 11% (n = 2056) of the fractures registered had the SFMA questionnaires completed at both baseline and after 1 year, and 10% of the EQ5d. Additionally, 52% of the PROM respondents were men, and the mean age of the PROM respondents was higher than the mean age of nonrespondents (51 years vs 38 years, *P* = <.001). The overall median SMFA arm/hand index score was 5 (IQR = 3) and EQ-5D 0.86 (IQR = 0.20) before the fracture and 10 (IQR = 13) and 0.85 (IQR = 0.2), respectively, after 1 year. The difference in changes in SMFA arm/hand index EQ-5D according to metacarpal, sex, treatment, and articular engagement is presented in Table 2. Fractures in the fifth metacarpal had a lower mean SMFA arm/hand function index change after 1 year compared with the other metacarpals. Women and surgically treated patients had a significantly larger increase in SMFA arm/hand function. However, no differences exceeded the MCID for the SMFA arm/hand index nor EQ-5D (8 and 0.1 points, respectively).

**Table 2:** Changes in EQ-5D and SMFA Arm/Hand Index Score 1 Year After Fracture

	EQā-5D Change Mean ± SD n = 1822	*P* Value	SMFA Arm/Hand Index Change Mean ± SD n = 2056	*P* Value
All fractures	−0.02 ± 0.20		5 ± 16	
Metacarpal				
First	−0.03 ± 0.23	.289∗	6 ± 18	< .001∗
Second	−0.02 ± 0.20		8 ± 20	
Third	−0.04 ± 0.20		9 ± 15	
Fouth	−0.00 ± 0.20		5 ± 15	
Fifth	−0.01 ± 0.20		4 ± 15	
Sex				
Men	−0.02 ± 0.21	.437†	4 ± 16	< .001†
Women	−0.02 ± 0.20		7 ± 16	
Treatment				
Nonsurgical	−0.01 ± 0.20	< .001†	4 ± 15	< .001†
Operative	−0.04 ± 0.21		7 ± 18	
Intra-articular fracture				
Yes	−0.01 ± 0.20	.001†	5 ± 16	.502†
No	−0.03 ± 0.24		5 ± 17	

EQ-5D, EuroQoL 5 dimensions.

∗ Kruskal-Wallis test.

† Mann-Whitney U test.

## Discussion

### Fracture epidemiology

There were differences in metacarpal fracture patterns between the sexes. We observed a bimodal age distribution in women and an unimodal distribution in men, which may be attributed to different risk factors and injury mechanisms between the sexes. Men are likely exposed to more risk factors in their working life, and women have a higher prevalence of osteoporosis later in life.^20^ Men accounted for the majority (70%) of the metacarpal fractures, which might reflect differences in activities, occupations, or other behavioral factors. This male proportion is in accordance with the report by Weum et al^6^ but somewhat less than that reported by Nakashian et al^9^ and Chung et al.^8^ This disparity might be explained by the number of included fractures or differences in the studied populations.

The anatomical distribution of fractures in the metacarpals showed notable variations. As in previous reports, the fifth metacarpal was the most frequently fractured, accounting for 57% of all metacarpal fractures, whereas the fourth, first, third, and second metacarpals followed in a descending order.^5^^,^^6^ Distal fractures were the most common in the fifth and second metacarpals, whereas shaft fractures were the most common in the third and fourth. This may be because the second and fifth metacarpal heads are more exposed and less stabilized by surrounding structures than the others. The anatomical distribution of fractures between the different metacarpals did not differ considerably between men and women (Table 1). However, differences between men and women were apparent in the anatomical distribution within each metacarpal, with distal fractures occurring more frequently in men and diaphyseal fractures being more prevalent in women. This might be explained by differing behavioral patterns, with punching injuries being more prevalent among men and torsional injuries, such as those caused by dog leashes, being more common among women. Anakwe et al^7^ found that fractures of the fifth metacarpal in males were associated with social deprivation, and this was explained by a higher prevalence of specific injury types, such as punching, in this group.

The fracture pattern differed between children and adults. Although a fracture of the fifth metacarpal was the most common across all age groups, children exhibited a higher prevalence of first metacarpal fractures compared with adults. This difference may be attributed to different activity patterns in children or different biomechanical factors in the child’s hands. Lempesis et al^10^ found a distribution like ours in 149 metacarpal fractures in children.

Fractures of the first metacarpal were more likely to be treated surgically. Men were treated surgically in 19% of all fractures, compared with 14% in women. High-energy injuries were more prevalent among men, potentially accounting for the higher occurrence of open injuries and more complex fractures necessitating surgical fixation in men. The mean age for men was lower than for women, and by speculation, younger patients may be more inclined to advocate for surgery because of cosmetic and functional requirements than older patients.

### PROMs

We did not find any relevant correlation between metacarpal fractures and self-reported function, health, or well-being of the patients in the register. There was a modest increase (ie, more disability) in SMFA arm/hand index 1 year after a metacarpal fracture. Women and surgically treated patients experienced larger increases in SMFA arm/hand scores. Fractures of the fifth metacarpal had a less negative effect on SMFA arm/hand index score compared with the other metacarpals. However, since the differences were small and did not exceed the MCID, the findings suggest that metacarpal fractures do not have a relevant impact on patient-rated hand function.

There was a very small decrease in EQ-5D scores (ie, worse) after 1 year, indicating no impact on quality of life by metacarpal fractures. There was no significant difference in mean EQ-5D index change after 1 year between the different metacarpals. There was a statistically significant difference in EQ-5D index scores between intra-articular and extra-articular fractures, as well as between surgical and nonsurgical treatment, but the differences were very small, and we regard them as clinically irrelevant.

### Strengths and limitations

The foremost strength of this study is its large sample size of 18,802 metacarpal fractures. Patients from all of Sweden are represented in the material, providing a good national overview. To our knowledge, this is the largest analysis of the epidemiology of metacarpal fractures to date. Earlier studies are either considerably smaller including 200–1569 fractures or large but lacking detailed analysis.^3^^,^^6^^,^^7^^,^^9^, ^10^, ^11^

One major limitation lies in the reliance on data sourced from a register with potentially incomplete or inaccurate recordings such as not coding for the exact location or injury mechanism. Although the SFR offers comprehensive overall national coverage, the coverage for hand fractures is lower compared with other fractures.^21^ All orthopedic departments in Sweden are affiliated with the SFR, and not all of them register hand fractures, which may impact data integrity. In some regions, less complicated fractures are treated at local emergency clinics staffed by general practitioners who do not report to the register. In Sweden, complicated hand fractures are often referred to as hand surgical centers, of which only two of seven contribute to SFR, introducing uncertainty regarding the proportion of surgically treated metacarpal fractures.

A general limitation of registry studies is that the selection of collected data are beyond the researcher’s control. The evaluation instruments, their timing, and other factors are decided by the registry holders and might not always suit your specific area of study.

The number of metacarpal fractures in children in this study is likely underreported, compared with other studies, because of how fractures in children are registered in the SFR, with a focus on physeal fractures of long bones. Hence, one must be careful not to draw far-fetched conclusions.^22^ However, we believe that the presented distribution of metacarpal fractures in children is reasonably correct.

Furthermore, the absence of information regarding specific nonsurgical treatment protocols, such as early mobilization, splinting, or casting, further compounds this limitation. There are no data on factors that could affect the decision to operate or not such as comorbidities, soft-tissue damage, and fracture dislocation in the SFR.

A limitation when analyzing the PROMs is the low response rate in patients, suggesting a risk for nonresponder bias. In total, 52% of the respondents were men, which is lower than expected since 70% of the metacarpal fractures affected men. Since the mean age of the PROM respondents was higher than the mean age of nonrespondents, young patients might be underrepresented in the PROM analysis. Juto et al^23^ compared nonresponders with responders in the SFR and found no significant difference between the groups regarding the preinjury survey and 1-year follow-up. Accordingly, Stirling et al^24^ have demonstrated that non-response does not need to result in bias. In all studies involving PROMs, recall bias may potentially influence the results. Within the SFR, patients are requested to retrospectively assess their baseline status, as it was approximately a week preceding the injury. Although this approach carries a risk of recall bias, it is hard to employ an alternative method in the context of injuries. Research on recall bias has shown that most PROMs are valid for the recall of patients’ health status up to 4 weeks before completing the forms.^25^

In conclusion, this study provides a comprehensive analysis of the distribution of metacarpal fractures and highlights the differences in the distribution and treatment between men and women.

## Conflicts of Interest

No benefits in any form have been received or will be received related directly to this article.
