# Supplementary material for: The Epidemiology of Metacarpal Fractures: A Descriptive Study Based on 18,802 Fractures From the Swedish Fracture Register
Source: J Hand Surg Glob Online. 2025 May 22;7(4):100725. doi: 10.1016/j.jhsg.2025.02.015 (PMC12150039; doi:10.1016/j.jhsg.2025.02.015)
Supplement: Supplementary Table 1 [file mmc1.docx]

**SUPPORTING INFORMATION**

**Supplementary table 1. Metacarpal fracture distribution in men and women.**

| **Metacarpal** | **Distal fractures**  **Men/Women**  **(n)** | **Distal Intraarticular fractures**  **Men/Women**  **(%)** | **Diaphyseal fractures**  **Men/Women**  **(n)** | **Proximal fractures**  **Men/Women**  **(n)** | **Proximal intraarticular fractures**  **Men/Women**  **(%)** |
| --- | --- | --- | --- | --- | --- |
| **1^st^** | **102/102** | **13/17** | **145/56** | **1043/366** | **53/44** |
| **2^nd^** | **420/133** | **21/21** | **231/117** | **150/87** | **67/53** |
| **3^rd^** | **173/71** | **21/23** | **560/321** | **220/81** | **47/47** |
| **4^th^** | **483/157** | **8/10** | **1237/722** | **448/176** | **58/36** |
| **5^th^** | **4109/1025** | **5/5** | **1757/993** | **1657/1021** | **62/49** |
